# Association between malnutrition and low tongue pressure in community-dwelling older people: a population-based cohort study

**DOI:** 10.1038/s41598-025-11229-x

**Published:** 2025-07-14

**Authors:** Ryota Takaoka, Keitaro Nishi, Maya Nakamura, Haruka Yoshinaga, Yusaku Noma, Yodai Hayashi, Sayaka Yuda, Yumiko Mishima, Momoko Ishikawa, Yusei Yanagita, Kouta Yamashiro, Kenichi Kume, Yuhei Matsuda, Takahiro Kanno, Hyuma Makizako, Toshihiro Takenaka, Takuro Kubozono, Mitsuru Ohishi, Tatsuo Okui

**Affiliations:** 1 Department of Maxillofacial Diagnostic and Surgical Science, Field of Oral and Maxillofacial Rehabilitation, Graduate School of Medical and Dental Sciences, Kagoshima University, Kagoshima, 890-8544 Japan; 2 Department of Oral and Maxillofacial Surgery, Kagoshima Prefectural Oshima Hospital, 1-18 Manatsu-cho, Amami, 894-0015 Kagoshima Japan; 3 Department of Oral and Maxillofacial Surgery, Shimane University Faculty of Medicine, 89-1 Enya-cho, Izumo, Shimane 693-8501 Japan; 4 Department of Physical Therapy, School of Health Sciences, Faculty of Medicine, Kagoshima University, Kagoshima, 890-8544 Japan; 5 Tarumizu Municipal Medical Center, Tarumizu Chuo Hospital, Kagoshima, 891-2124 Japan; 6 Department of Cardiovascular Medicine and Hypertension, Graduate School of Medical and Dental Sciences, Kagoshima University, Kagoshima, 890-8544 Japan

**Keywords:** Tongue pressure, Malnutrition, Longitudinal study, Community-dwelling older people, Propensity score matching, Malnutrition, Geriatrics, Risk factors, Epidemiology, Gerodontics

## Abstract

The aim of this study was to analyze the data from a cohort study of community-dwelling older people to clarify the impact of low tongue pressure on the onset of malnutrition. The analysis was divided into a baseline and a longitudinal analysis. The baseline analysis included 765 people (median age: 74 years; 276 men and 489 women), and the longitudinal analysis included 100 people (median age: 72 years; 33 men and 67 women). The baseline analysis showed that tongue pressure and the diagnosis of low tongue pressure using a cut-off of 30 kPa were associated with the risk of malnutrition (tongue pressure: odds ratio [OR] = 0.972, 95% confidence interval [95% CI] = 0.953–0.992, *p* = 0.006; the diagnosis of low tongue pressure: OR = 1.555, 95% CI = 1.050–2.302, *p* = 0.027). In the longitudinal analysis, tongue pressure and the diagnosis of low tongue pressure at baseline were associated with the onset of the risk of malnutrition after 4 years (tongue pressure: OR = 0.952, 95% CI = 0.907–0.998, *p* = 0.041; the diagnosis of low tongue pressure: OR = 2.698, 95% CI = 1.148–6.341; *p* = 0.023). In community-dwelling older people, low tongue pressure could increase the risk of future malnutrition.

**Supplementary Information:**

The online version contains supplementary material available at 10.1038/s41598-025-11229-x.

## Introduction

 Malnutrition in older people is a major international concern due to aging populations in various countries. Estimates suggest that approximately 25% of the global older population (aged ≥ 65 years) is currently malnourished or at risk of malnutrition^1^. An association between malnutrition and a number of health outcomes, such as frailty, muscle wastage, hypothermia, osteoporosis, mood changes and cognitive impairment, has been demonstrated in older people^2^. Furthermore, malnutrition has been shown to directly contribute to poor health outcomes, such as increased mortality^2^. These direct health effects also contribute to increased medical costs, further increasing the burden on society^3^. The identification of risk factors for malnutrition and subsequent intervention is thus a great medical and social challenge.

Malnutrition is thought to be caused by a combination of complex factors, including not only inappropriate dietary intake but also diseases such as malignant tumors and chronic inflammation, as well as social factors^4–7^. The Global Leadership Initiative on Malnutrition (GLIM) criteria were proposed in 2018 by major clinical nutrition societies around the world as a standard malnutrition diagnosis that encompasses these factors^8^. Malnutrition diagnosis using the GLIM criteria consists of three steps: initial screening for the risk of malnutrition, diagnosis of malnutrition in cases identified through screening, and assessment of severity. The first step, screening for malnutrition risk, aims to identify individuals at risk of malnutrition from a large number of individuals, and the use of the Mini Nutritional Assessment Tool - Short Form (MNA-SF), Malnutrition Universal Screening Tool (MUST) and Nutritional Risk Screening 2002 (NRS-2002) is recommended for this purpose^8^. Screening for malnutrition risk is a crucial step in preventing poor prognosis and reducing healthcare costs, particularly among community-dwelling older people.

Research into the association between a decline in oral function and the onset and progression of malnutrition is a hot topic, and there has already been a report on the association between malnutrition and oral hypofunction in a cross-sectional study of community-dwelling older people^9^ and a prospective cohort study of older people who regularly receive health checks at hospitals^10^. Among oral functions, tongue pressure is an indicator that can quantitatively and objectively evaluate the function of the tongue, which is closely related to eating and swallowing functions. Tongue pressure has been adopted as one of the diagnostic criteria for oral hypofunction^11^ and sarcopenic dysphagia in Japan^12^ and research on the relationship with health outcomes is currently being actively pursued. Previous studies showed that tongue pressure decreases with age^13^ that a positive correlation is observed between tongue pressure and MNA score^14^ and that low tongue pressure is associated with malnutrition in elderly individuals^14–17^. However, as the above-mentioned studies were cross-sectional in design, no findings have yet been obtained regarding the longitudinal causal relationship between tongue pressure and malnutrition in community-dwelling older people.

Our hypothesis is that low tongue pressure is a risk factor for malnutrition. If this causal relationship is confirmed, it is expected that approaches such as tongue pressure training will contribute to the prevention of malnutrition in older people. The aim of this study is to analyze the effect of low tongue pressure on the risk of malnutrition assessed using the MNA-SF, using data from the Tarumizu Study, a prospective cohort study of community-dwelling older people.

## Methods

### Study design and participants

In this study, we adopted a prospective cohort study design, with exposure as tongue pressure and outcome as malnutrition risk. Participants in this study were enrolled from among those who participated in the Tarumizu City health assessment survey (Tarumizu Study) for people aged ≥ 40 years, conducted by Kagoshima University, Tarumizu City Hall and Tarumizu Chuo Hospital between July and December 2018. In the Tarumizu study, the city hall recruited participants by sending public information leaflets to residents’ addresses by post. This study conforms to the STROBE guidelines for prospective cohort studies, and approval was obtained from the Ethics Committee on Epidemiological and its related Studies, Sakuragaoka Campus, Kagoshima University (Approval No. 170351) prior to the start of the study. Participants provided written informed consent prior to study participation. Also, all methods were performed in accordance with Declaration of Helsinki. In this study, after investigating the cross-sectional association between tongue pressure and malnutrition risk in 2018 as a baseline analysis, we investigated the association between tongue pressure in 2018 and malnutrition risk in 2022 as a longitudinal analysis, targeting participants who had normal nutritional status at baseline. The inclusion criteria for the baseline analysis were set at participants aged ≥ 65 years who participated in the 2018 Tarumizu study. The exclusion criteria were set in accordance with past reports, and in order to ensure the reliability of the tongue pressure measurement results and the questionnaire, as follows: (1) participants aged < 65 years; (2) data missing; (3) dementia; (4) cerebrovascular disease^18^; (5) hemiplegia; and (6) Parkinson’s disease^19^. The inclusion criteria for the longitudinal analysis were set at participants aged ≥ 65 years who participated in the 2018 and 2022 Tarumizu studies. Exclusion criteria were set as follows: (1) participants aged < 65 years; (2) data missing; (3) dementia; (4) cerebrovascular disease; (5) hemiplegia; (6) Parkinson’s disease; and (7) participants assessed as being at risk of malnutrition as of 2018. Participants in the longitudinal analysis were followed up for four years, and outcomes were assessed in the Tarumizu study, which was conducted between July 2022 and March 2023.

### Assessment of tongue pressure

The JMS tongue pressure measuring device (TPM-01; JMS Corporation, Hiroshima, Japan) was used to measure tongue pressure. Subjects placed the balloon probe in their mouth and fixed the plastic pipe by biting lightly on the center of the bilateral central incisors. They then closed their lips, raised their tongue and pressed the probe against the hard palate with maximum force; tongue pressure was recorded by the examiner. Measurements were taken three times, and the maximum value was recorded. This recorded tongue pressure was used in the analysis as a continuous variable. In addition, based on the evaluation criteria for oral hypofunction proposed by the Japanese Society of Gerodontology, low tongue pressure was diagnosed as a tongue pressure of less than 30 kPa^11^. Using this cutoff, the participants were divided into a normal tongue pressure group and low tongue pressure group.

### Assessment of malnutrition

The Mini Nutritional Assessment Short-Form (MNA-SF) was used for nutritional assessment. The MNA-SF is a simple malnutrition screening tool developed for older people^20,21^. The MNA-SF was developed as a simplified version of the MNA, reducing the number of questions from 18 in the original version to 6, and is widely used as a simple and practical malnutrition screening tool requiring only 5 min per test. The MNA-SF consists of 6 questions from A to F. Each question is rated on a score of 0 to 3 points, and the final total for the 6 items is calculated. The highest total score is 14 and the lowest is 0. The lower the score, the higher the risk of malnutrition. Either lower leg circumference (CC) or body mass index (BMI) is used for item F. In the present study, BMI was used to assess item F. A person was assessed as being at risk of malnutrition if the total score was ≤ 11 points^22^.

### Background data

We sampled the following variables as background data: sex (male/female), age (years), years of schooling (years), living alone (none/alone), sleep duration (hours per day), drinking frequency (frequency per week), smoking (none/having a history/current smoker), energy intake (calculated from brief-type self-administered diet history questionnaire [BDHQ]: kcal per day), diabetes mellitus (none/having a history/under treatment), cancer (none/having a history/under treatment), sarcopenia (diagnosis based on Asian Working Group for Sarcopenia [AWGS 2019]: none/sarcopenia/severe sarcopenia)^23^.

### Statistical analysis

Only subjects with complete data were entered into the present analysis; subjects with missing values for the variables of exposure, outcome, or background data were excluded. The missing data were not imputed. Instead, the variables were compared between the groups before and after excluding the missing values to assess whether there were any changes in characteristics between the groups. Descriptive statistics were used to summarize the data, with the median (IQR: interquartile range) used for continuous variables and the absolute value (%: percentages) for categorical variables. For tests of normality, the Kolmogorov–Smirnov test was used when the number of samples in a group was more than 100, and the Shapiro–Wilk test was used when the number was less than 100. Mann-Whitney U test and Pearson’s chi-square test were used for between-group comparisons in each variable. For risk analysis in the baseline population, we used univariate and multivariate binomial logistic regression analyses to assess the association between the risk of malnutrition and tongue pressure and between the risk of malnutrition and the diagnosis of low tongue pressure in the 2018 data. The variables for multivariate analysis were input using the forced entry method for those that had been reported in the past as confounding factors and for which no multicollinearity could be confirmed using the variance inflation factor (VIF). Variables with VIF < 7 were determined not to be multicollinear. The independent variables entered were sex, age, years of education, living alone, drinking frequency, smoking, sleep duration, energy intake, diabetes mellitus, cancer, sarcopenia, tongue pressure and the diagnosis of low tongue pressure. Propensity score matching was used in the longitudinal analysis to adjust for the same potentially confounding variables as in the baseline analysis. To calculate the estimated propensity score for each participant, we used a binary logistic regression analysis with the diagnosis of low tongue pressure as the dependent variable and sex, age, years of schooling, living alone, drinking frequency, smoking, sleep duration, energy intake, diabetes mellitus, cancer, and sarcopenia as the independent variables. The discriminate power of the propensity scores was quantified by measuring the receiver-operating-characteristic area (c-statics), and it was judged that appropriate discrimination was possible if the c-statics was within the range of 0.6–0.9. Using the estimated propensity score, nearest neighbor matching was performed so that the ratio of subjects in the normal tongue pressure group to the low tongue pressure group was 1:1. The balance before and after matching was evaluated using the standardized mean difference (SMD), and if it was 0.1 or less, it was evaluated as being well balanced. The caliper was set to the standard deviation of the estimated propensity score × 0.2. After adjustment by propensity score matching, the association between tongue pressure in 2018 and risk of malnutrition in 2022, and the association between a diagnosis of low tongue pressure in 2018 and risk of malnutrition in 2022 were assessed using Mann-Whitney U test, Pearson’s chi-square test and univariate logistic regression analysis. To account for the possibility that unknown confounding factors could overturn the significant results of the study, E-values were calculated against odds ratios and confidence intervals for the results of the logistic regression analysis of the baseline and longitudinal analyses^24,25^. We performed sensitivity analyses to evaluate the robustness of the results: in the baseline analysis, we conducted multivariate logistic regression analysis using directed acyclic graphs to select the minimum number of confounding factors from background data; in the longitudinal analysis, we conducted multivariate logistic regression analysis using directed acyclic graphs, as well as propensity score-adjusted multivariate logistic regression analysis. Statistical analyses were performed using statistical software (SPSS ver29; IBM, Armonk, NY, USA; and R version 4.4.2). Two-sided p-values were calculated for all analyses, with a significance level of *p* < 0.05.

## Results

### Demographic characteristics and group comparison

**Fig. 1:**
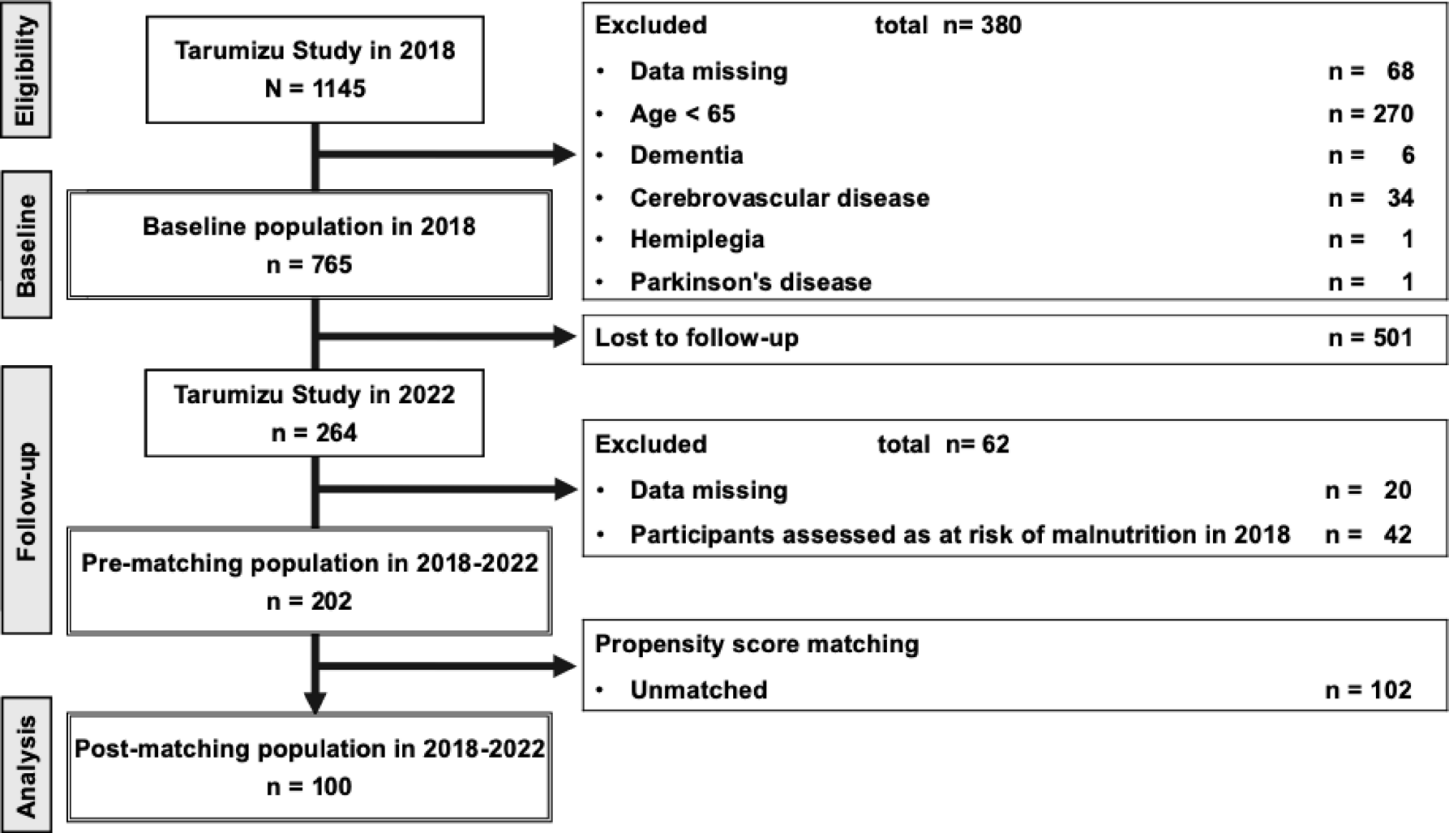
Flow diagram for participants included in this study.

The participant selection process is shown in Fig. 1. Of the 1145 participants in the 2018 Tarumizu Study, a total of 765 eligible subjects were included in the baseline analysis, and then propensity score matching was performed on 202 subjects who were not at risk of malnutrition at baseline and could be followed up until 2022, resulting in 100 subjects who were ultimately included in the longitudinal analysis. Table S1 shows the changes in the characteristics of the subjects before and after excluding the missing data. No variables were significantly different between before and after the exclusion of missing data. The characteristics of the subjects in the baseline analysis are shown in Table 1, and the characteristics of the subjects before and after propensity score matching in the longitudinal analysis are shown in Table 2. The distributions of propensity scores before and after matching are shown in Fig. S1. The baseline analysis included 765 participants. Among them, the number in the low tongue pressure group was 276 (36.1%), and the number in the group at risk of malnutrition was 147 (19.2%). The percentage of people at risk of malnutrition was 15.7% in the normal tongue pressure group and 25.4% in the low tongue pressure group. Based on the background data, participants in the low tongue pressure group were more likely to be female, older, and living alone and more likely to have sarcopenia than those in the normal tongue pressure group. Before matching, the study population consisted of 202 participants. Among them, 60 (29.7%) participants were categorized into the low tongue pressure group, and 70 (34.7%) participants developed malnutrition risk between 2018 and 2022. The percentage of participants who developed malnutrition risk was 30.3% in the normal tongue pressure group and 45.0% in the low tongue pressure group. Comparison of the background data between the normal tongue pressure group and low tongue pressure group showed significant differences in drinking frequency, but no interpretable trend was seen. For the 202 subjects in the pre-matching population, the diagnosis of low tongue pressure was used as the dependent variable, and the estimated propensity score was calculated using sex, age, years of schooling, living alone, drinking frequency, smoking, sleep duration, energy intake, diabetes mellitus, cancer and sarcopenia as the independent variables. The c statistic for the estimated propensity score was 0.727 (Fig. S2), with a standard deviation of 0.1782. The caliper was set to 0.2. Nearest-neighbor matching was performed at a ratio of 1:1 with a caliper value of 0.0356. Matching excluded 102 participants and 100 were finally included in the post-matching population. Among the 100 participants in the post-matching population, 50 (50.0%) were categorized into the low tongue pressure group, and 35 (35.0%) developed malnutrition risk between 2018 and 2022. The participants who developed malnutrition risk made up 24.0% of the normal tongue pressure group and 46.0% of the low tongue pressure group. Although the background data revealed no significant difference in any of the variables between the normal and the low tongue pressure groups, the standardized mean difference (SMD) analysis revealed some items with incomplete balance adjustment, such as living alone, smoking, sleep duration, diabetes mellitus, cancer, sarcopenia (0.1 ≤ SMD < 0.2), and drinking frequency (0.3 ≤ SMD <  0.4).

**Table 1:** Demographic characteristics and group comparisons by the diagnosis of low tongue pressure in a baseline analysis.

Variables	Categories	Total (*n* = 765)	Normal tongue pressure group (*n* = 489)	Low tongue pressure group (*n* = 276)	*P*-value	SMD
		Median [IQR], *n* (%)	Median [IQR], *n* (%)	Median [IQR], *n* (%)		
Sex	Male	276 (36.1)	187 (38.2)	89 (32.2)	0.021*	0.126
	Female	489 (63.9)	302 (61.8)	187 (67.8)		
Age	Years	74 [70–80]	73 [69–77]	76 [71–82]	0.034*	0.463
Years of schooling	Years	12[9 − 12]	12[9–12]	11 [9–12]	0.089	0.134
Living alone	Alone	265 (34.6)	155(31.7)	110 (39.9)	0.002*	0.171
	None	500 (65.4)	334 (68.3)	166 (60.1)		
Drinking frequency	None	457 (59.7)	271 (55.4)	186 (67.4)	0.504	0.321
	Less than weekly	41 (5.4)	27 (5.5)	14 (5.0)		
	Once a week	14 (1.8)	11 (2.2)	3 (1.1)		
	Twice a week	25 (3.3)	17 (3.5)	8 (2.9)		
	3 times a week	23 (3.0)	14 (2.9)	9 (3.3)		
	4 times a week	9 (1.2)	5 (1.0)	4 (1.4)		
	5 times a week	21 (2.7)	12 (2.5)	9 (3.3)		
	6 times a week	16 (2.1)	13 (2.7)	3 (1.1)		
	Every day	159 (20.8)	119 (24.3)	40 (14.5)		
Smoking	None	530 (69.3)	327 (66.9)	203 (73.6)	0.256	0.147
	Smoking history	197 (25.8)	136 (27.8)	61 (22.1)		
	Current smoker	38 (5.0)	26 (5.3)	12 (5.3)		
Sleep duration	Less than 4 h	34 (4.4)	24 (4.9)	10 (3.6)	0.079	0.073
	4–6 h	317 (41.4)	201 (41.1)	116 (42.0)		
	6–8 h	320 (41.8)	206 (42.1)	114 (41.3)		
	More than 8 h	94 (12.3)	58 (11.9)	36 (13.0)		
Energy intake	kcal/day	1871.6 [1556.4-2236.1]	1872.2 [1567.7-2212.4]	1866.9 [1538.6-2314.9]	0.650	0.080
Diabetes mellitus	None	653 (85.4)	417 (85.3)	236 (85.5)	0.879	0.064
	Having a history	8 (1.0)	4 (0.8)	4 (1.4)		
	Under treatment	104 (13.6)	68 (13.9)	36 (13.0)		
Cancer	None	673 (88.0)	423 (86.5)	250 (90.6)	0.367	0.129
	Having a history	72 (9.4)	52 (10.6)	20 (7.2)		
	Under treatment	20 (2.6)	14 (2.9)	6 (2.2)		
Sarcopenia	None	662 (86.5)	444 (90.8)	218 (79.0)	< 0.001*	0.336
	Sarcopenia	73 (9.5)	33 (6.7)	40 (14.5)		
	Severe sarcopenia	30 (3.9)	12 (2.5)	18 (6.5)		
Nutritional status in 2018	Normal	618 (80.8)	412 (84.3)	206 (74.6)	0.001*	0.240
	At risk of malnutrition	147 (19.2)	77 (15.7)	70 (25.4)		

All variables indicate data collected in 2018. The p-value was calculated from a comparison of the normal tongue pressure group and the low tongue pressure group. IQR: interquartile range. SMD: standardized mean difference. *p < 0.05.

**Table 2:** Demographic characteristics and group comparisons between pre- and post-matching by the diagnosis of low tongue pressure in longitudinal analysis.

Variables	Categories	Pre-matching population (*n* = 202)				Post-matching population (*n* = 100)			
		Normal tongue pressure group (n = 142)	Low tongue pressure group (n = 60)	P-Value	SMD	Normal tongue pressure group (n = 50)	Low tongue pressure group (n = 50)	P-Value	SMD
		Median [IQR], n (%)	Median [IQR], n (%),			Median [IQR], n (%)	Median [IQR], n (%)		
Sex	Male	65 (45.8)	21 (35.0)	0.157	0.114	17 (34.0)	16 (32.0)	0.832	0.043
	Female	77 (54.2)	39 (65.0)			33 (66.0)	34 (68.0)		
Age	Years	72[69–76]	72[70–77]	0.299	0.051	73[69–79]	72[70–76]	0.833	0.089
Years of schooling	Years	12[10.75-13]	12[9–14]	0.435	0.060	12[9–12]	12[9–14]	0.942	0.091
Living alone	Alone	46 (32.4)	16 (26.7)	0.264	0.126	10 (20.0)	13 (26.0)	0.476	0.143
	None	96 (67.6)	44 (73.3)			40 (80.0)	37 (74.0)		
Drinking frequency	None	69 (48.6)	32 (53.3)	0.046*	0.638	28 (56.0)	31 (62.0)	0.823	0.345
	Less than weekly	12 (8.5)	6 (10.0)			3 (6.0)	4 (8.0)		
	Once a week	4 (2.8)	0 (0)			0 (0)	0 (0)		
	Twice a week	2 (1.4)	5 (8.3)			2 (4.0)	1 (2.0)		
	3 times a week	6 (4.2)	1 (1.7)			2 (4.0)	1 (2.0)		
	4 times a week	1 (0.7)	1 (1.7)			0 (0)	1 (2.0)		
	5 times a week	3 (2.1)	4 (6.7)			2 (4.0)	3 (6.0)		
	6 times a week	7 (4.9)	0 (0)			0 (0)	0 (0)		
	Every day	38 (26.8)	11 (18.3)			13 (26.0)	9 (18.0)		
Smoking	None	90 (63.4)	45 (75.0)	0.235	0.272	37 (74.0)	38 (76.0)	0.828	0.123
	Smoking history	42 (29.6)	11 (18.3)			10 (20.0)	8 (16.0)		
	Current smoker	10 (7.0)	4 (6.7)			3 (6.0)	4 (8.0)		
Sleep duration	Less than 4 h	5 (3.5)	2 (3.3)	0.914	0.112	1 (2.0)	1 (2.0)	0.942	0.126
	4–6 h	70 (49.3)	32 (53.3)			24 (48.0)	27 (54.0)		
	6–8 h	57 (40.1)	21 (35.0)			20 (40.0)	18 (36.0)		
	More than 8 h	10 (7.0)	5 (8.3)			5 (10.0)	4 (8.0)		
Energy intake	kcal/day	1903.0 [1584.7-2312.6]	1836.6 [1534.4-2342.1]	0.519	0.107	1840.8 [1620.3-2237.1]	1802.2 [1469.6-2407.6]	0.694	0.015
Diabetes mellitus	None	120 (84.5)	52 (86.7)	0.693	0.062	43 (86.0)	45 (90.0)	0.538	0.123
	Having a history	0 (0)	0 (0)			0 (0)	0 (0)		
	Under treatment	22 (15.5)	8 (13.3)			7 (14.0)	5 (10.0)		
Cancer	None	124 (87.3)	53 (88.3)	0.136	0.298	45 (90.0)	46 (92.0)	0.842	0.118
	Having a history	15 (10.6)	3 (5.0)			3 (6.0)	3 (6.0)		
	Under treatment	3 (2.1)	4 (6.7)			2 (4.0)	1 (2.0)		
Sarcopenia	None	133 (93.7)	55 (91.7)	0.632	0.158	45 (90.0)	47 (93.5)	0.461	0.148
	Sarcopenia	8 (5.6)	5 (8.3)			5 (10.0)	3 (6.0)		
	Severe sarcopenia	1 (0.7)	0 (0)			0 (0)	0 (0)		
Nutritional status in 2022	Normal	99 (69.7)	33 (55.0)	0.045*	0.307	38 (76.0)	27 (54.0)	0.021*	0.474
	At risk of malnutrition	43 (30.3)	27 (45.0)			12 (24.0)	23 (46.0)		

The data for the nutritional status variable were collected in 2022, and the data for all other variables were collected in 2018. The p-value was calculated from a comparison of the normal tongue pressure group and the low tongue pressure group. IQR: interquartile range. SMD: standardized mean difference. *p < 0.05.

### Association between tongue pressure and risk of malnutrition in a baseline population

The results of the Pearson’s Chi-squared test are shown in Table 1, and the results of the Mann-Whitney U test are shown in Table 3. In the group comparison, the proportion of participants at risk of malnutrition was higher in the low tongue pressure group (*p* = 0.001), and also tongue pressure was lower in the malnutrition risk group (*p* < 0.001).

**Table 3:** Mann-Whitney U test for comparison of tongue pressure by nutritional status group in the baseline population.

Variables	Normal nutritional status group	At risk of malnutrition group	*P*-Value
	Median [IQR]	Median [IQR]	
Tongue pressure (kPa)	33.6 [27.6–39.4]	30.9 [22.9–37.1]	< 0.001*

The dependent variable was set as tongue pressure in 2018, and the independent variable was set as nutritional status in 2018. IQR: interquartile range. *p < 0.05.

### Risk analysis of tongue pressure and risk of malnutrition in baseline population

The results of the univariate and multivariate logistic regression analysis are shown in Table 4. Univariate logistic regression analysis showed that both tongue pressure and the diagnosis of low tongue pressure were associated with the risk of malnutrition (tongue pressure: OR = 0.965, 95% CI = 0.948–0.983, *p* < 0.001; the diagnosis of low tongue pressure: OR = 1.818, 95% CI = 1.263–2.617, *p* = 0.001). Similarly, in multivariate logistic regression analysis, tongue pressure and the diagnosis of low tongue pressure were both associated with the risk of malnutrition (tongue pressure: OR = 0.972, 95% CI = 0.953–0.992, *p* = 0.006, E-value = 1.13, CI = 1.07; the diagnosis of low tongue pressure: OR = 1.555, 95% CI = 1.050–2.302, *p* = 0.027, E-value = 1.8, CI = 1.18).

**Table 4:** Logistic regression analysis in the baseline population.

Variables	Univariate			Multivariate		
	Odds ratio	95% CI	*P*-Value	Odds ratio	95% CI	*P*-Value
Model 1						
Sex	1.586	1.068–2.356	0.022*	1.848	0.95–3.595	0.070
Age	1.030	1.001–1.060	0.039*	0.970	0.936–1.006	0.105
Years of schooling	0.931	0.862–1.006	0.072	0.980	0.898–1.070	0.651
Living alone	0.563	0.390–0.812	0.002*	1.554	1.025–2.355	0.038*
Drinking frequency	1.049	0.991–1.111	0.098	0.990	0.919–1.066	0.783
Smoking	0.841	0.606–1.168	0.302	1.227	0.760–1.981	0.403
Sleep duration	1.091	0.860–1.384	0.472	1.001	0.777–1.290	0.993
Energy intake	1.000	1.000–1.000	0.664	1.000	1.000–1.000	0.795
Diabetes mellitus	1.044	0.808–1.350	0.740	1.102	0.842–1.443	0.479
Cancer	0.975	0.634–1.501	0.909	1.049	0.664–1.655	0.839
Sarcopenia	2.748	1.990–3.793	< 0.001*	2.827	1.959–4.080	< 0.001*
Tongue pressure	0.965	0.948–0.983	< 0.001*	0.972	0.953–0.992	0.006*
Model 2						
Sex	1.586	1.068–2.356	0.022*	1.877	0.960–3.670	0.066
Age	1.030	1.001–1.060	0.039*	0.975	0.941–1.011	0.174
Years of schooling	0.931	0.862–1.006	0.072	0.974	0.892–1.063	0.549
Living alone	0.563	0.390–0.812	0.002*	1.541	1.017–2.336	0.041*
Drinking frequency	1.049	0.991–1.111	0.098	0.992	0.921–1.068	0.827
Smoking	0.841	0.606–1.168	0.302	1.236	0.764–2.001	0.388
Sleep duration	1.091	0.860–1.384	0.472	1.014	0.788–1.305	0.913
Energy intake	1.000	1.000–1.000	0.664	1.000	1.000–1.000	0.815
Diabetes mellitus	1.044	0.808–1.350	0.740	1.098	0.839–1.435	0.497
Cancer	0.975	0.634–1.501	0.909	1.043	0.660–1.648	0.856
Sarcopenia	2.748	1.990–3.793	< 0.001*	2.802	1.942–4.042	< 0.001*
Diagnosis of low tongue pressure	1.818	1.263–2.617	0.001*	1.555	1.050–2.302	0.027*

In models 1 and 2, the dependent variable was set as nutritional status in 2018. CI: confidence interval. *p < 0.05.

### Association between tongue pressure and risk of malnutrition in the pre- and post-matching population

The results of the Pearson’s Chi-square test are shown in Table 2, and the results of the Mann-Whitney U test are shown in Table 5. In both the pre- and post-matching populations, the proportion of individuals who would be at risk of malnutrition in 2022 was higher in the low tongue pressure group than the normal tongue pressure group in 2018 (pre-matching population: *p* = 0.045; post-matching population: *p* = 0.021), and correspondingly, the participants with malnutrition risk in 2022 had lower tongue pressure in 2018 (pre-matching population: *p* = 0.014; post-matching population: *p* = 0.018).

**Table 5:** Mann-Whitney U test for comparison of tongue pressure by nutritional status group in the pre- and post-matching populations.

Variables	Pre-matching population			Post-matching population		
	Normal nutritional status group	At risk of malnutrition group	P-value	Normal nutritional status group	At risk of malnutrition group	P-value
	Median [IQR]	Median [IQR]		Median [IQR]	Median [IQR]	
Tongue pressure (kPa)	36.4 [29.9–41.1]	31.2 [27.4–38.7]	0.014*	32.8 [26.1–39.7]	28.2[25.2–31.2]	0.018*

The dependent variable was set as tongue pressure in 2018, and the independent variable was set as nutritional status in 2022. IQR: interquartile range. *p < 0.05.

### Risk analysis of tongue pressure and risk of malnutrition in the post-matching population

The results of the univariate logistic regression analysis are shown in Table 6. Logistic regression analysis showed that both tongue pressure and the diagnosis of low tongue pressure were associated with the risk of malnutrition. An association was also found between the diagnosis of low tongue pressure in 2018 and risk of malnutrition in 2022 (tongue pressure: OR = 0.952, 95% CI = 0.907–0.998, *p* = 0.041; E-value = 1.18, CI = 1.03; the diagnosis of low tongue pressure: OR = 2.698, 95% CI = 1.148–6.341, *p* = 0.023; E-value = 2.67, CI = 1.35).

**Table 6:** Univariate logistic regression analysis in the post-matching population.

Variables	Odds ratio	95%CI	*P*-Value
Tongue pressure	0.952	0.907–0.998	0.041*
Diagnosis of low tongue pressure	2.698	1.148–6.341	0.023*

The dependent variable was set as the nutritional status in 2022. CI: confidence interval. *p < 0.05.

### Sensitivity analysis

To assess the robustness of the results, a logistic regression was conducted using a directed acyclic graph as a sensitivity analysis at baseline (Table S2). In the longitudinal study, we conducted two sensitivity analyses: a multivariate logistic regression analysis with a directed acyclic graph (Table S3), and a propensity score-adjusted multivariate logistic regression analysis (Table S4). The results of the sensitivity analysis for both the baseline and longitudinal studies were similar to the results of the main analysis.

## Discussion

The major finding of this study is that low tongue pressure may be a risk factor for the onset of malnutrition in community-dwelling older people. In this study, we followed the community-dwelling elderly who participated in a health examination in Japan and analyzed the effect of tongue pressure at baseline on the risk of later malnutrition. Previously, our research group investigated the association between oral function decline and geriatric syndromes such as frailty, sarcopenia, mild cognitive impairment, and low protein intake in community-dwelling older people^26–28^. Although there have been many reports on the association between oral function and geriatric syndromes, the longitudinal causal relationship between tongue pressure and malnutrition has been unclear. This study showed that a decrease in tongue pressure and a diagnosis of low tongue pressure were significantly associated with later onset of malnutrition, and the results of the sensitivity analysis were similar to the main analysis. Thus our hypothesis that low tongue pressure is a risk factor for malnutrition is likely to be correct, and our results were valid.

In general, tongue pressure is defined as the maximum pressure when the tongue is pressed against the palate. This measured pressure is thought to be generated by the coordinated action of intrinsic and extrinsic tongue muscles, and is positioned as one of the diagnostic criteria for sarcopenic dysphagia as a measure of muscle strength in swallowing-related muscles^12^. Epidemiologically, tongue pressure decreases with age^13^. As to a possible gender difference, the findings are mixed: a meta-analysis found no gender difference in tongue pressure in individuals ≥ 60 years old, but a significantly lower tongue pressure in women among individuals < 60 years old^29^. Anatomically, atrophy of the tongue^30^ and geniohyoid muscles^31^ have been reported in older people compared to younger controls. Histologically, atrophy of the lingual muscles is frequently seen in older rats compared to younger controls^32^ and the muscle fiber size of the intrinsic and extrinsic tongue muscles of rats has been reported to decrease with age^33^ while in humans, amyloid deposition in the tongue has been reported to increase with age in those aged ≥ 60 years^34^. In the baseline population in the present study, the low tongue pressure group had more women and was older. In older people who are relatively healthy, such as those living in a community, it is possible that a gender difference in tongue pressure, reflecting the difference in muscle strength between men and women, persists even past ≥ 60 years of age. Regarding age, the characteristics of the baseline population in this study were consistent with previous reports showing an association between aging and low tongue pressure. Although the mechanism by which low tongue pressure leads to malnutrition is still unknown, we speculate that it may be a three-step process involving dysphagia. First, low tongue pressure causes problems with food-bolus formation and transport to the pharynx during the oral preparation phase of swallowing. Second, dysphagia prolongs the mealtime, causing the dysphagic eater to become exhausted before they have eaten enough. Alternatively, dysphagia causes repeated aspiration pneumonia. Third, the risk of malnutrition increases due to the inability to secure sufficient food over a long period of time, or due to the exhaustion caused by aspiration pneumonia. In fact, a low tongue pressure has been correlated with dysphagia in many clinical settings, including healthy older people^35,36^ stroke patients^37–39^ and older people admitted to hospital without neurological disorders^40^. Other studies have found that the relationship between dysphagia and malnutrition is interdependent^41,42^. Finally, low tongue pressure has been associated with prolonged mealtimes and an increased incidence of pneumonia^43–45^. Collectively, these results suggest that low tongue pressure affects the nutritional status of older people via dysphagia.

Two minor findings of this study also bear mention. First, in the baseline multivariate analysis, there was an association between living alone and the risk of malnutrition. In this study, we included years of education and living alone as measurable items, based on previous reports of an association between these social factors and malnutrition^4^. According to the report, living alone is associated with malnutrition/malnutrition risk at an odds ratio of around 1.8, and the results of this study were similar. Although social factors are not included in the nutrition screening tools and GLIM criteria currently used internationally, for analyses limited to community-dwelling older people, including living alone as a diagnostic item may improve the ability to predict malnutrition. On the other hand, there was no association between education years and malnutrition risk. In Japan, compulsory education guarantees a certain level of education, and this could have kept education years from being reflected in the results.

Secondly, we found that sarcopenia and tongue pressure were independently associated with malnutrition risk. It is already widely known that sarcopenia and malnutrition are mutually associated^46^ and as mentioned above, there are multiple reports of an association between tongue pressure and malnutrition. However, many of the previous reports have suggested that sarcopenic dysphagia, a condition that shares low tongue pressure and sarcopenia, is associated with malnutrition, so our finding that tongue pressure was directly associated with malnutrition independently of sarcopenia is of value. However, the relationship between the three variables of sarcopenia, tongue pressure and nutritional status, which were seen as independent factors, could not be verified in this study. Future investigations using structural equation modelling will be needed to examine the causal relationship among these three variables.

This study had three main limitations. First, the observation period was limited to four years. Because the number of years required for malnutrition to develop in older community-living individuals has not been clearly determined, we cannot rule out that our observation period was too short. Second, there was a bias in the selection of subjects. Our subjects were selected at a health examination with a recruitment format, which could have biased the selection toward healthy volunteers. In addition, the COVID-19 pandemic forced a significant reduction in the scale of the Tarumizu study from 2019 onwards, resulting in 65.5% of cases being lost to follow-up. This would have considerably affected our results. Third, there was a limitation with respect to the adjustments for confounding. This study is a single cohort study in a single region of Japan, and the analysis uses complete data with no missing values. Furthermore, even after propensity score matching, the balance adjustment was not sufficient for some variables. Due to these limitations, the generalizability of the results may be limited. However, in terms of the observation period, the incidence of malnutrition risk over the four-year period of this study was 34.7%, which is broadly similar to the 32% prevalence of malnutrition risk in community-dwelling older people in the previous study^22^ so it is considered to be a sufficient research period. In addition, although we did not perform imputation for missing data, there was no significant difference in the distribution of variables between the complete data and the data before missing data were excluded. Furthermore, in the matching, the results of the sensitivity analysis conducted on the pre-matching group were similar to the results of the main analysis, and it is thought that the impact of excluding missing data and matching on the characteristics of the analysis population was not significant.

This study suggests that low tongue pressure may be a risk factor for malnutrition in older people living in the community, and that detecting a decrease in tongue pressure may enable malnutrition to be detected at an earlier stage. In other words, tongue pressure screening tests could uncover malnutrition risk in older community-living people at an earlier stage, and thereby improve the health of the population. Preventing malnutrition also has significant socioeconomic benefits, as it can lead to reduced medical costs and a reduced burden on medical institutions. In addition, continuous screening would help to maintain good nutritional status and promote health education in the community, leading to long-term health improvement. Based on the above, the tongue pressure test is an extremely useful tool for preventing malnutrition risk and managing nutritional status in community-dwelling groups of older individuals, and it is hoped that it will be actively used from the perspective of the population strategy.

## Conclusions

In community-dwelling older individuals (age ≥ 65 years), tongue pressure at baseline was associated with malnutrition risk four years later.

## Electronic supplementary material

Below is the link to the electronic supplementary material.

Supplementary Material 1

## Data Availability

The data presented in this study are available on request from the corresponding author.
