# Supplementary material for: Association between malnutrition and low tongue pressure in community-dwelling older people: a population-based cohort study
Source: Sci Rep. 2025 Jul 14;15:25420. doi: 10.1038/s41598-025-11229-x (PMC12260005; doi:10.1038/s41598-025-11229-x)
Supplement: Supplementary file 1 — Supplementary Material 1 [file 41598_2025_11229_MOESM1_ESM.pdf]

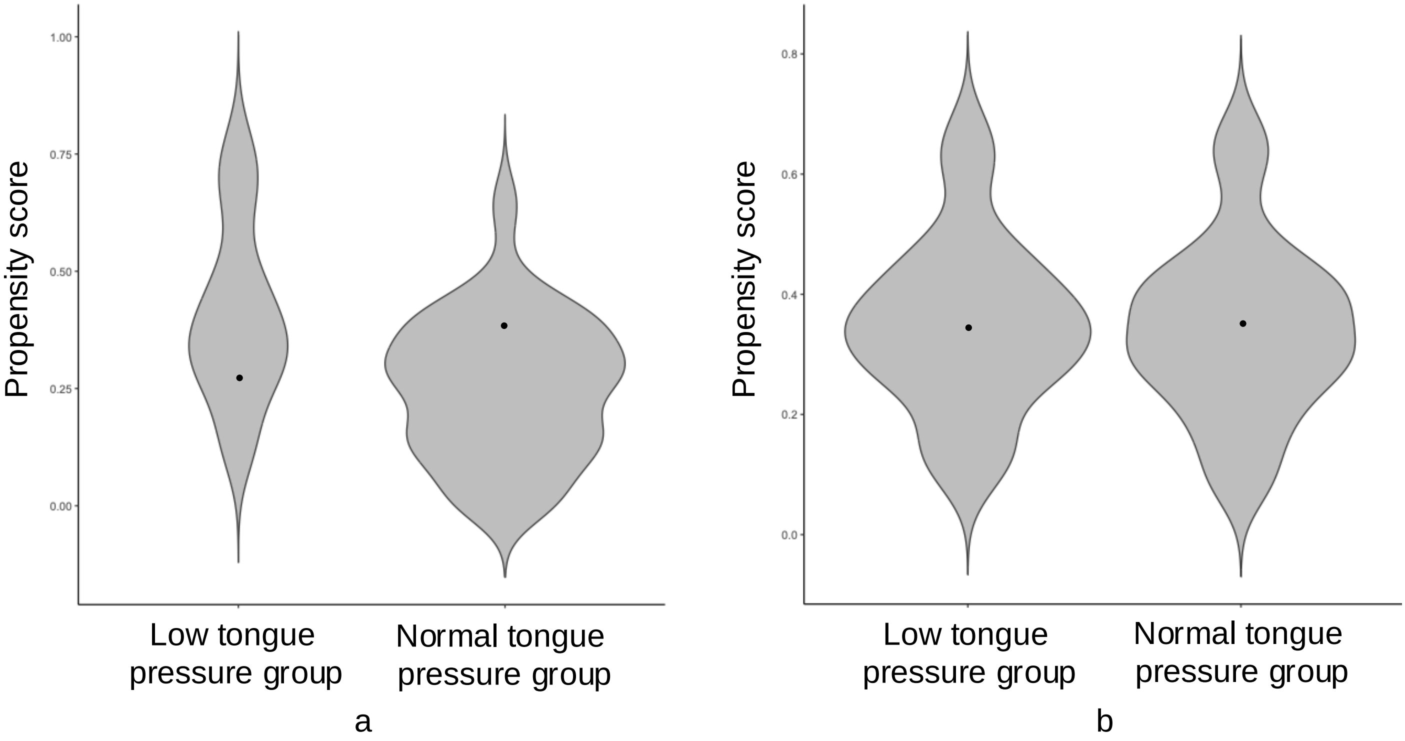

Figure S1. Distribution of propensity scores in the pre- and post-matching population

a: Pre-matching population. b: Post-matching population. •: median.

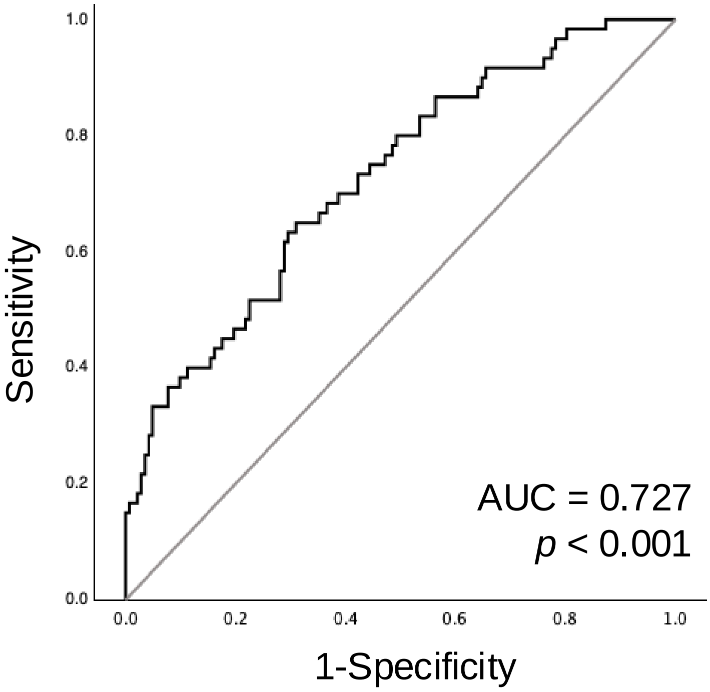

Figure S2. ROC analysis for calculating c statics

The dependent variable was the diagnosis of low tongue pressure in 2018, and the independent variable was the estimated propensity score. AUC: area under the curve.

Table S1. Participant characteristics and group comparisons in the complete data and the data before excluding missing values

Variables	Categories	Complete data (n = 765) median [IQR], n (%)	Complete + missing data(n = 833) median [IQR], n (%)	P-Value	SMD
**Sex**	Male	276 (36.1)	297 (35.7)	0.860	0.009
	Female	489 (63.9)	536 (64.3)		
**Age**	Years	74 [70-80]	74 [70-80]	0.619	0.054
**Years of schooling**	Years	12[9−12]	12[9−12]	0.837	0.008
**Living alone**	Alone	265 (34.6)	287 (34.5)	0.993	<0.001
	None	500 (65.4)	542 (65.1)		
**Drinking frequency**	None	457 (59.7)	494 (59.3)	1.000	0.040
	Less than weekly	41 (5.4)	51 (6.1)		
	Once a week	14 (1.8)	14 (1.7)		
	Twice a week	25 (3.3)	27 (3.2)		
	3 times a week	23 (3.0)	27 (3.2)		
	4 times a week	9 (1.2)	9 (1.1)		
	5 times a week	21 (2.7)	23 (2.8)		
	6 times a week	16 (2.1)	16 (1.9)		
	Every day	159 (20.8)	172 (20.6)		
**Smoking**	None	530 (69.3)	579 (69.5)	0.995	0.005
	Smoking history	197 (25.8)	213 (25.6)		
	Current smoker	38 (5.0)	41 (5.0)		
**Sleep duration**	Less than 4 hours	34 (4.4)	37 (4.4)	0.985	0.019
	4 - 6 hours	317 (41.4)	351 (42.1)		
	6 - 8 hours	320 (41.8)	342 (41.1)		
	More than 8 hours	94 (12.3)	99 (11.9)		
**Energy intake**	kcal/day	1871.6 [1556.4-2236.1]	1863.5 [1549.2-2223.8]	0.717	0.021
**Diabetes mellitus**	None	653 (85.4)	716 (86.0)	0.932	0.019
	Having a history	8 (1.0)	9 (1.0)		
	Under treatment	104 (13.6)	108 (13.0)		
**Cancer**	None	673 (88.0)	730 (87.6)	0.977	0.011
	Having a history	72 (9.4)	81 (9.7)		
	Under treatment	20 (2.6)	22 (2.6)		
**Sarcopenia**	None	662 (86.5)	684 (82.1)	0.986	0.008
	Sarcopenia	73 (9.5)	77 (9.2)		
	Severe sarcopenia	30 (3.9)	32 (3.8)		
**Tongue pressure**	kPa	33.3 [26.6-39.0]	33.3 [26.7-38.8]	0.960	0.005
**Diagnosis of low tongue pressure**	Normal tongue pressure	489 (63.9)	528 (63.4)	0.948	0.003
	Low tongue pressure	276 (36.1)	296 (35.5)		
**Nutritional status in 2018**	Normal	618 (80.8)	642 (77.1)	0.846	0.012
	At risk of malnutrition	147 (19.2)	148 (17.8)		

All variables indicate data collected in 2018. The p-value was calculated from a comparison of complete data and complete + missing data.
IQR: interquartile range. SMD: standardized mean difference. *$p<0.05$.

Table S2. Baseline sensitivity analysis: multivariate logistic analysis using directed acyclic graphs to select independent variables

Model 1				Model 2			
**Variables**	**Odds ratio**	**95% CI**	**P-Value**	**Variables**	**Odds ratio**	**95% CI**	**P-Value**
Sex	1.640	1.085-2.479	0.070	Sex	1.676	1.109-2.532	0.014*
Age	0.982	0.949-1.015	0.105	Age	0.987	0.956-1.020	0.443
Sarcopenia	2.818	1.966-4.040	<0.001*	Sarcopenia	2.806	1.958-4.020	<0.001*
Tongue pressure	0.973	0.953-0.992	0.006*	Diagnosis of low tongue pressure	1.545	1.049-2.276	0.028*

In Models 1 and 2, the dependent variable was set to the nutritional status in 2018. CI: confidence interval. *p<0.05.

Table S3. Longitudinal sensitivity analysis: multivariate logistic analysis using directed acyclic graphs to select independent variables

Model 1				Model 2			
**Variables**	**Odds ratio**	**95% CI**	**P-Value**	**Variables**	**Odds ratio**	**95% CI**	**P-Value**
Sex	1.016	0.558-1.851	0.959	Sex	1.022	0.561-1.862	0.942
Age	0.972	0.915-1.033	0.364	Age	0.981	0.925-1.042	0.535
Sarcopenia	1.727	0.619-4.818	0.297	Sarcopenia	1.692	0.608-4.710	0.314
Tongue pressure	0.966	0.934-1.000	0.047*	Diagnosis of low tongue pressure	1.894	1.010-3.551	0.046*

In Models 1 and 2, the dependent variable was set to the nutritional status in 2022. CI: confidence interval. *p<0.05.

Table S4. Longitudinal sensitivity analysis: propensity score-adjusted logistic regression analysis

Model 1				Model 2			
**Variables**	**Odds ratio**	**95% CI**	**P-Value**	**Variables**	**Odds ratio**	**95% CI**	**P-Value**
Propensity score	0.478	0.084-2.728	0.406	Propensity score	0.370	0.061-2.254	0.281
Tongue pressure	0.965	0.932-0.999	0.042*	Diagnosis of low tongue pressure	2.201	1.107-4.375	0.024*

In Models 1 and 2, the dependent variable was set to the nutritional status in 2022. CI: confidence interval. *p<0.05.
